# First Description of Sulphur-Oxidizing Bacterial Symbiosis in a Cnidarian (Medusozoa) Living in Sulphidic Shallow-Water Environments

**DOI:** 10.1371/journal.pone.0127625

**Published:** 2015-05-26

**Authors:** Sylvie Abouna, Silvina Gonzalez-Rizzo, Adrien Grimonprez, Olivier Gros

**Affiliations:** 1 Institut de Biologie Paris-Seine, UMR 7138—Evolution Paris-Seine, Equipe Biologie de la Mangrove. Université des Antilles et de la Guyane, UFR des Sciences Exactes et Naturelles, Département de Biologie, BP 592. 97159 Pointe-à-Pitre cedex, Guadeloupe, France; 2 C_3_MAG, UFR des Sciences Exactes et Naturelles, Université des Antilles et de la Guyane, BP 592. 97159 Pointe-à-Pitre, Guadeloupe (French West Indies); Universite Pierre et Marie Curie, FRANCE

## Abstract

**Background:**

Since the discovery of thioautotrophic bacterial symbiosis in the giant tubeworm *Riftia pachyptila*, there has been great impetus to investigate such partnerships in other invertebrates. In this study, we present the occurrence of a sulphur-oxidizing symbiosis in a metazoan belonging to the phylum Cnidaria in which this event has never been described previously.

**Methodology/Principal Findings:**

Scanning Electron Microscope (SEM), Transmission Electron Microscope (TEM) observations and Energy-dispersive X-ray spectroscopy (EDXs) analysis, were employed to unveil the presence of prokaryotes population bearing elemental sulphur granules, growing on the body surface of the metazoan. Phylogenetic assessments were also undertaken to identify this invertebrate and microorganisms in thiotrophic symbiosis. Our results showed the occurrence of a thiotrophic symbiosis in a cnidarian identified as *Cladonema* sp.

**Conclusions/Significance:**

This is the first report describing the occurrence of a sulphur-oxidizing symbiosis in a cnidarian. Furthermore, of the two adult morphologies, the polyp and medusa, this mutualistic association was found restricted to the polyp form of *Cladonema* sp.

## Introduction

With over 13,000 living species of cnidarians identified, they are vast groups of marine invertebrates belonging to the phylum Cnidaria living mostly in marine shallow water ecosystems [1–3]. The phylum of Cnidaria is subdivided into six classes: Anthozoa that comes in the polyp form (such as anemones, corals), the recently reclassified Myxozoa (parasitic animals), Cubozoa, Hydrozoa, Scyphozoa, and Staurozoa [2–6].

The production of two adult morphologies, either in the shape of polyps or medusae (such as jellyfish), is intrinsic features of cnidarians. Furthermore, in many invertebrates belonging to this phylum, both forms are generated alternately during their life history [1, 7–9]. A typical life cycle in colonial or solitary cnidarians begins with a planula, a pear-shaped ciliated larva developing from gamete fusion, upon spawning of male and female medusae freely in the water column. The planula after anchorage on a hard substratum, metamorphoses into a primary polyp which gives rise, asexually to new polyps and so forth [10–11]. Once conditions are favourable, the polyp via metagenesis will then generate and release a multitude of medusae, that will adopt later a pelagic mode of life and a new cycle starts, invariably [1, 8, 9, 12–15].

Generally, cnidarians are carnivorous creatures that prey to meet their nutritional demands. Nevertheless, some members of this phylum can take advantage of symbiosis, to complement their nutrient intake. Corals and sea anemones in close partnership with endosymbiotic dinoflagellate algae (genus S*ymbiodinium*, commonly termed zooxanthellae), illustrate perfectly this alternative, whereby symbionts can provide via transfer, up to 60% of their photosynthate to their hosts [2, 16–26]. Other authors have also reported comparable processes in the green *Hydra* sp. with their endosymbiotic photosynthetic algae of genus *Chlorella*. [22, 27–29] and in the jellyfish *Cassiopea xamachana* residing in the mangrove [30], in which the dinoflagellate symbionts met up to 169% of the jellyfish’s nutritional requirements.

Like photosynthetic symbiosis, sulphur-oxidizing symbiosis has also been proven to play a substantial role in the nutrition (ranging from 100% in organisms without digestive tract as for *Riftia pachyptila*, to lower in other marine invertebrates possessing a reduced digestive tract) of their hosts by providing them with organic compounds biosynthesized from inorganic carbon [31]. Symbiotic sulphur-oxidizing bacteria are chemoautotrophs that possess the ability to make use of sulphide or other inorganic reduced sulphur as electron donors to generate energy in the form of ATP via oxidative phosphorylation. This serves to fuel carbon dioxide fixation, via Calvin cycle or reverse tricarboxylic acid cycle, into organic compounds, ultimately providing the host with metabolites essential for its own needs. Usually, oxygen is the final electron acceptor of this electron transport chain system [32–38]. Protection against sulphide poisoning has also been suggested as an alternative role of sulphur-oxidizing bacteria to their hosts [39–41]. Hydrogen sulphide is a strong cytochrome c oxidase inhibitor which hinders the functioning of the respiratory chain. However, whether these bacteria truly detoxify host’s microenvironment, remains contentious [42].

In various sulphidic environments, thioautotrophic bacteria can organize as free-living organisms in bacterial mats [43–46], or in chemosynthetic symbioses with diverse marine invertebrates colonizing those habitats [34]. This kind of mutualistic relationship was described later in other reducing habitats, namely shallow waters, cold seeps [47, 48], sea-grass beds [49], whale falls [43] sunken woods [50, 51], and organic rich mud or mangrove peat [36]. Investigations of these extreme environments have also revealed their unexpected biodiversity. Importantly, a common denominator to all these dissimilar extreme environments, is the presence of an inverse gradient of oxygen and sulphide (or methane), so-called chemocline. It is at its oxic-anoxic interface that chemosynthetic symbioses prosper [32, 33, 37].

In marine mangrove, comparable chemoclines exist due to a steady production of biogenic sulfidic molecules from the sediment or wood falls [51]. In this habitat, sulphur-oxidizing microorganisms were found in symbiosis with the lucinid bivalve *Lucina pectinata* [52], the ciliate *Zoothamnium niveum* [36, 50, 53], the nematode *Eubostrichus dianae* [54] and pseudovorticellids [50]. Recently, we observed in this same aquatic biotope, a kind of mutualism involving sulphur-oxidizing bacteria that had not been reported before [55]. Here we describe for the first time, the occurrence of a symbiosis between thioautotrophic bacteria and a cnidarian (Medusozoa), *Cladonema* sp. living in sulphidic shallow-water environments.

## Materials and Methods

### Sampling site

Individuals of *Cladonema* sp. were collected by hand from the marine sediment of the mangrove lagoon “Manche à Eau” (61°33’24”W-16°16’36”N) in Guadeloupe, French West Indies, Caribbean. Only seven polyps were found at 3 years interval close to sunken leaves of *Rhizophora mangle* covering the sediment. The first two individuals collected in 2008 were used for SEM and TEM studies. The five other individuals collected in July 2011, were used for EDXs analysis and SEM observations, DNA extraction and phylogenetic analyses (including the bacterial phylogeny). Three medusa individuals were collected from sea grass beds of *Caulerpa racemosa* from the same lagoon in January 2013.

No specific permissions were required from these locations/activities. Our study did not involve endangered or protected species.

### Ultrastructural analyses

Samples collected were observed and photographed with a stereomicroscope before preparation for Scanning Electron Microscope (SEM) and Transmission Electron Microscope (TEM) observations in order to detect possible symbiosis.

Samples for SEM observations were fixed 2 hours at 4°C in 2% glutaraldehyde solution in cacodylate buffer (900mOsm, pH 7.2). They were then dehydrated in graded concentrations of acetone, critical point dried in CO_2_ and sputter-coated with gold before observation with a FEI Quanta 250 at 20kV. For TEM observations, samples were prefixed for one hour at 4°C in 2.5% glutaraldehyde in 0.1M pH 7.2 cacodylate buffer adjusted to 900mOsm. After a brief rinse in the same buffer, they were fixed for 45 minutes at room temperature in 1% osmium tetroxide in the same buffer with a final osmolarity adjusted to 1000mOsm, then rinsed in distilled water and post-fixed with 2% aqueous uranyl acetate for one more hour at RT before embedding in epoxy resin and observation in a Leo 912 TEM at 120Kv.

### Energy-dispersive X-ray spectroscopy (EDXs) analysis

In order to detect elemental compounds from individuals analyzed (such as sulphur), freshly fixed samples (as described for SEM preparation samples above) were observed using an Environmental Scanning Electron Microscope (FEI Quanta 250) operating at 5 kV under an environmental pressure of 7 Torrs at 5°C. EDX spectra were obtained using a M-max 500 mm^2^ Oxford detector.

### DNA extraction and amplification

DNA from Polyps was extracted by disrupting cells via three repeated cycles of freezing (5min at -80°C) and thawing (5min at 90°C) to release DNA. DNA from medusa stage was extracted using DNeasy Blood and Tissue kit (Qiagen) according to the manufacturer’s instructions.

The genes coding for 16S rRNA and 18S rRNA were amplified using universal primer set: 8F/907R (~907bp) for bacterial 16S rRNA gene (8F: 5’agagtttgatcctggctcag3’ and 907R: 5’ccgtcaattcmtttragttt3’) [56] and 1F/5R primer set (~950bp) (1F: 5’tacctgggttgatcctgccagtag3’ and 5R: 5’cttggcaaatgctttcgc3’) [57] for eukaryotic18S rRNA gene. PCR amplifications were performed as follows: 94°C for 5 min, 30 cycles of 94°C 30 s, 52°C 30 s, 72°C 90 s and finally 72°C for 10 min.

Specific primers targeting the 16S bacterial sequences obtained from polyp DNA were designed manually (16S_polyp_ectosym_1F: 5’ggactgagaggtcgaacagc3’ and 16S_polyp_ectosym1R: 5’gcgtcagtattggtccaggt3’) in order to verify the presence of polyp sulphur-oxidizing symbiont in medusa’s DNA. For this set of specific primers, the PCR conditions were performed as follows: 94°C for 5 min, 30 cycles of 94°C 30 s, 58°C 30 s, 72°C 1min and finally 72°C for 7 min.

### Gene cloning and sequencing

Amplicons of 18S rRNA gene of medusa and polyp were directly sequenced by Genoscreen (http://www.genoscreen.com).

On the other hand, the prokaryotic 16S rRNA gene sequences were first purified using QIAquick PCR purification kit (Qiagen) and then cloned with pGEM-T cloning kit (promega) according to manufacturer’s instruction. Inserts from 13 positive clones were isolated and sequenced by Genoscreen (http://www.genoscreen.com) using the vector primers T7 and SP6. Following analysis, the 18S rRNA and 16S rRNA gene sequences obtained in this study were then deposited in the GenBank database under no. KJ493943 and KJ493944 respectively.

### Phylogenetic analysis of 16S rRNA and 18S rRNA gene sequences

The gene sequences encoding 18S and 16S rRNA obtained (926 bp and 895 bp respectively) from polyps following analysis, were compared with the National Center of Biotechnology information NCBI (http://www.ncbi.nlm.nih.gov) database using BLAST [58]. Best hits were included in phylogenetic analyses. The phylogenetic analyses were conducted using MEGA version 5 [59]. Sequences aligned using MUSCLE and alignments were checked manually. The phylogenetic trees were constructed from multiple-aligned data using the Maximum Likelihood (ML) method with Tamurai-Nei as genetic distance model. Nodes robustness was assessed by performing 1000 bootstrap replicates, and only bootstrap values above 50% are indicated at the nodes of the trees.

## Results

### Morphological and ultrastructural analysis of the benthic polypoid phase

**Fig 1:**
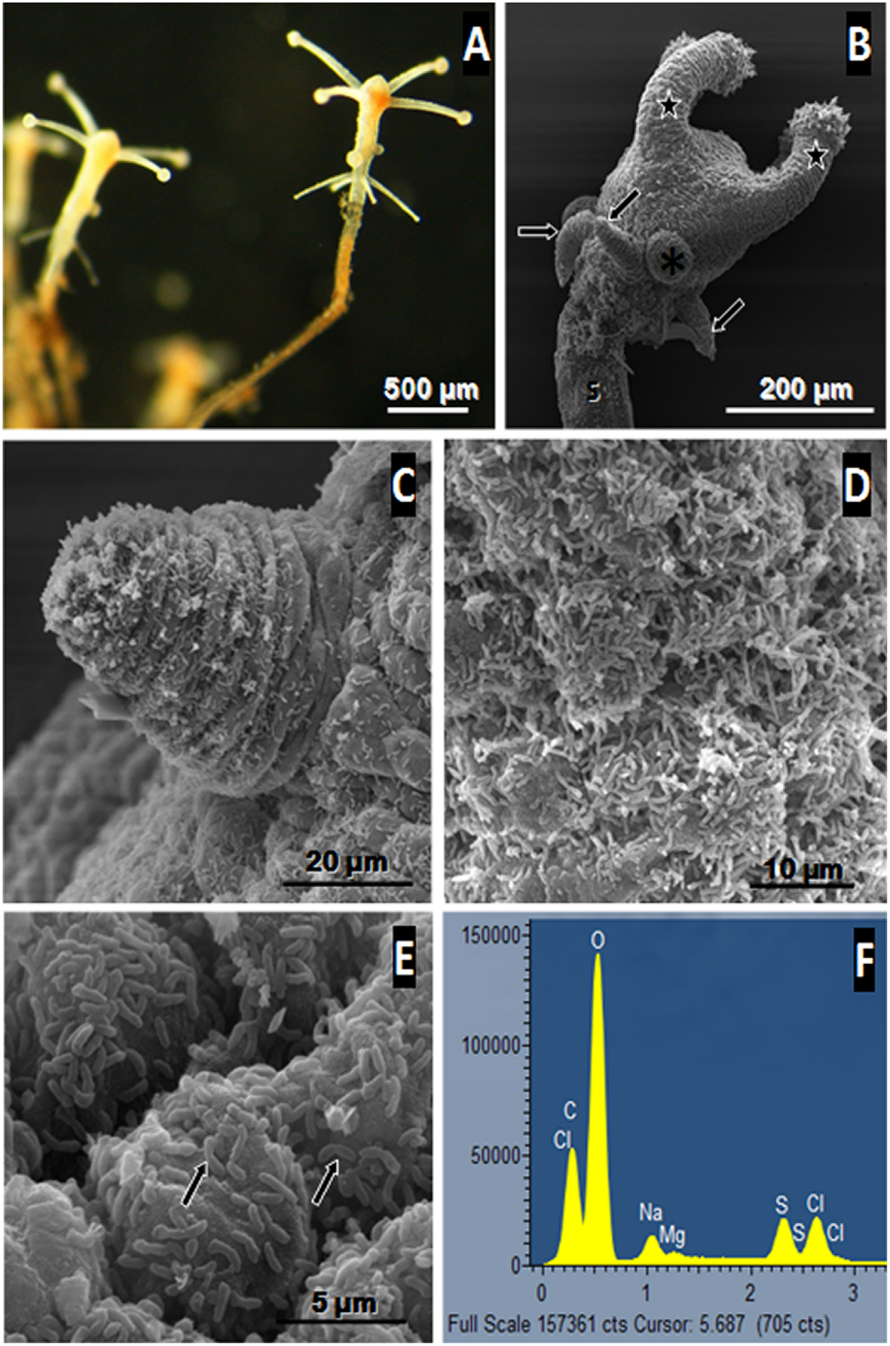
Structural analysis of polyp stage of *Cladonema* sp. (A) light micrograph of freshly collected polyps connected by a tube-like hydrocauli. The polyps appear white in colour especially for the tentacles. SEM image (B) shows that the polyp is composed of an entocodon (asterisk), a stalk (S), capitate (star) and filiform tentacles (arrows). A higher magnification of the polyp displays a filiform tentacle and small bacteria that can be clearly distinguished at the surface of the polyp (C). D and E show ectosymbiotic rod-shaped bacteria covering the polyp. Some of these are dividing (arrows) suggesting a high metabolism. The EDX spectrum obtained from the polyp (F) shows a peak of elemental sulphur suggesting that such bacteria are thioautotrophic (Cl: chloride, Na: sodium, Mg: magnesium, C: carbon, O: oxygen, S: elemental sulphur).

**Fig 2:**
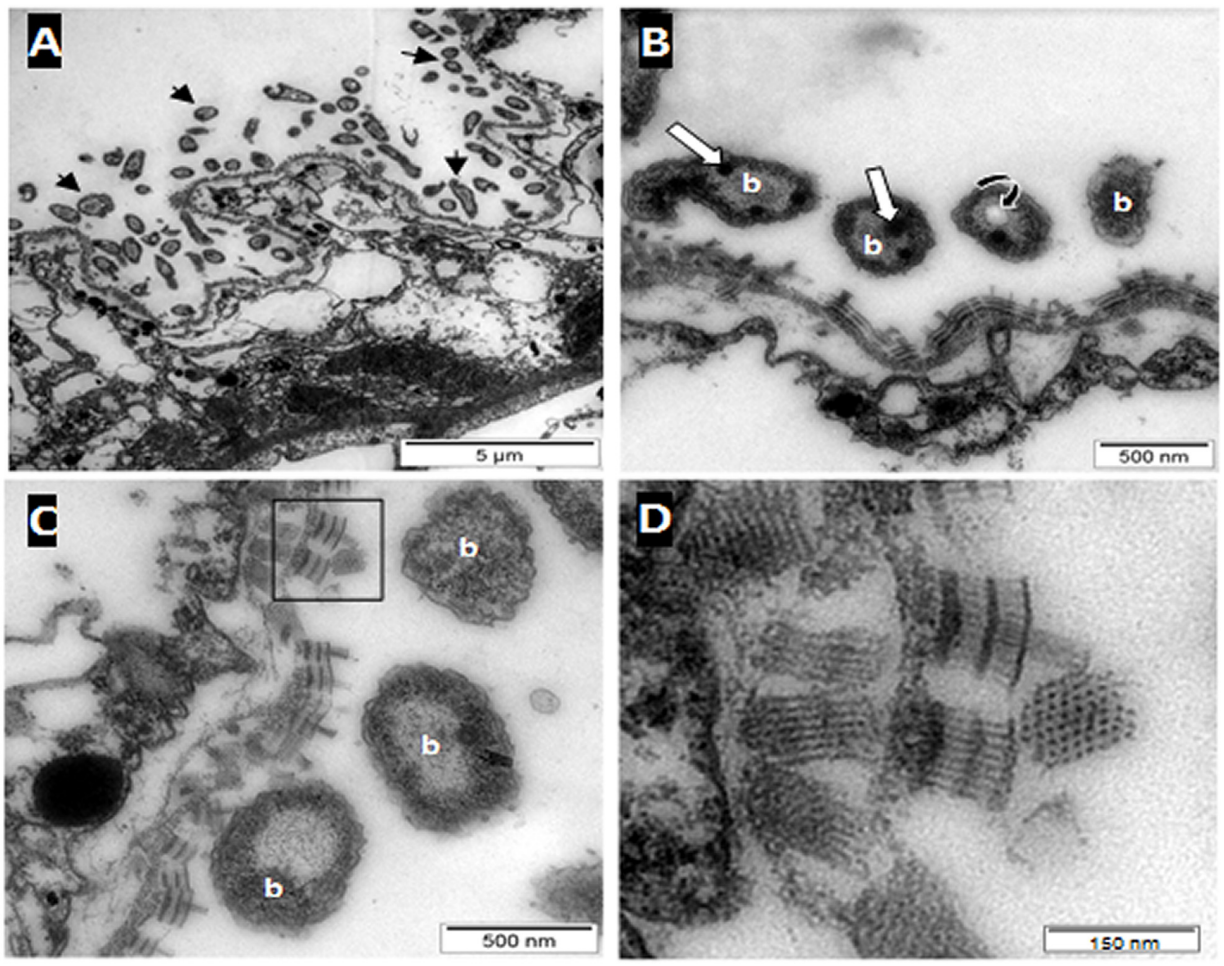
Ultrastructural analysis of polyp by TEM. Thin sections clearly show that the bacteria (arrows) are distributed exclusively on the body surface of polyps. No intracellular bacteria could be observed suggesting that there is no endosymbiosis in *Cladonema* sp. Higher magnification of the ectosymbionts (B) shows that the cytoplasm of the bacteria located outside the host tissue (b) contained two kinds of inclusions. The non-membrane-bound inclusions correspond to glycogen-like granules (white arrows) distributed throughout the cytoplasm while empty membrane-bound inclusions (curved arrow) correspond to sulphur granules probably located within the periplasm. The ectosymbionts appear to be fixed to the host cytoplasmic membrane (C) though atypical structures (see inset). Fig D displays a higher magnification of an atypical structure which is organized on two levels of “tubes” with a central tuft in contact with the bacteria. The nature of such “tubes” is unknown.

The individuals collected, formed delicate branched colonies containing few polyps (up to five for the sample collected in 2011) and were connected by tube-like hydrocauli (Fig 1A). The latter was connected by a small horizontal root-like stolon that anchored the colony into the sediment. Such structure was found between numerous sunken leaves of *Rhizophora mangle* covering the marine mangrove sediment. The colony was small and the polyps themselves were tiny and characterized by a white color (Fig 1A). These polyps are roughly cylindrical surrounded by four tentacles at the terminal end (Fig 1A and 1B). According to SEM views, the whole body of each polyp appeared to be covered by small bacteria (up to 1μm in length) forming a bacterial coat that sometimes seemed relatively dense (Fig 1C and 1D). These ectosymbiotic bacteria could divide at the surface of the polyps suggesting that they were in a productive environment allowing bacterial growth (Fig 1E). In order to characterize the metabolic type of the bacterial symbionts involved in such association, two polyps were analyzed in EDXs using an environmental SEM searching for elemental compound [46, 60]. The analysis showed that sulphur was one of the main elements detected from the bacteria (Fig 1F), thus suggesting that these prokaryotic cells contained elemental sulphur granules which are a typical feature of sulphur-oxidizing bacteria. Sulphur was also detected using Raman spectrometry (55) from the same polyp-type. So both techniques allowed the detection of elemental sulphur from the polyps. Furthermore, analyses of thin sections of the specimen observed by TEM, showed that bacteria were localized only outside the host tissue and no intracellular bacteria or endosymbiosis could be observed in *Cladonema* sp. (Fig 2A). The bacteria were only distributed at the surface of the polyp body and presented the typical double membrane of gram negative bacteria. Moreover, the bacterial cytoplasm contained membrane-bound empty vesicles which corresponded in fact to sulphur granules located within the periplasmic space (Fig 2B). The bacterial cytoplasm also contained numerous non membrane-bound irregular inclusions that are considered to be glycogenic storage granules. Most of the bacterial cell volume was occupied by the nucleoid area characterized by tenuous DNA filaments while the ribosomes were close to the internal membrane (Fig 2B). Ectosymbionts appeared to be attached to the host cytoplasmic membrane via a particular structure (Fig 2C). The structure seemed to be composed of three layers of dense tubules (30 nm in length) with an external central brush (Fig 2D) in contact with the outer membrane of the ectosymbiont (Fig 2C).

### Phylogenetic analysis

#### Polyp *Cladonema* sp

**Fig 3:**
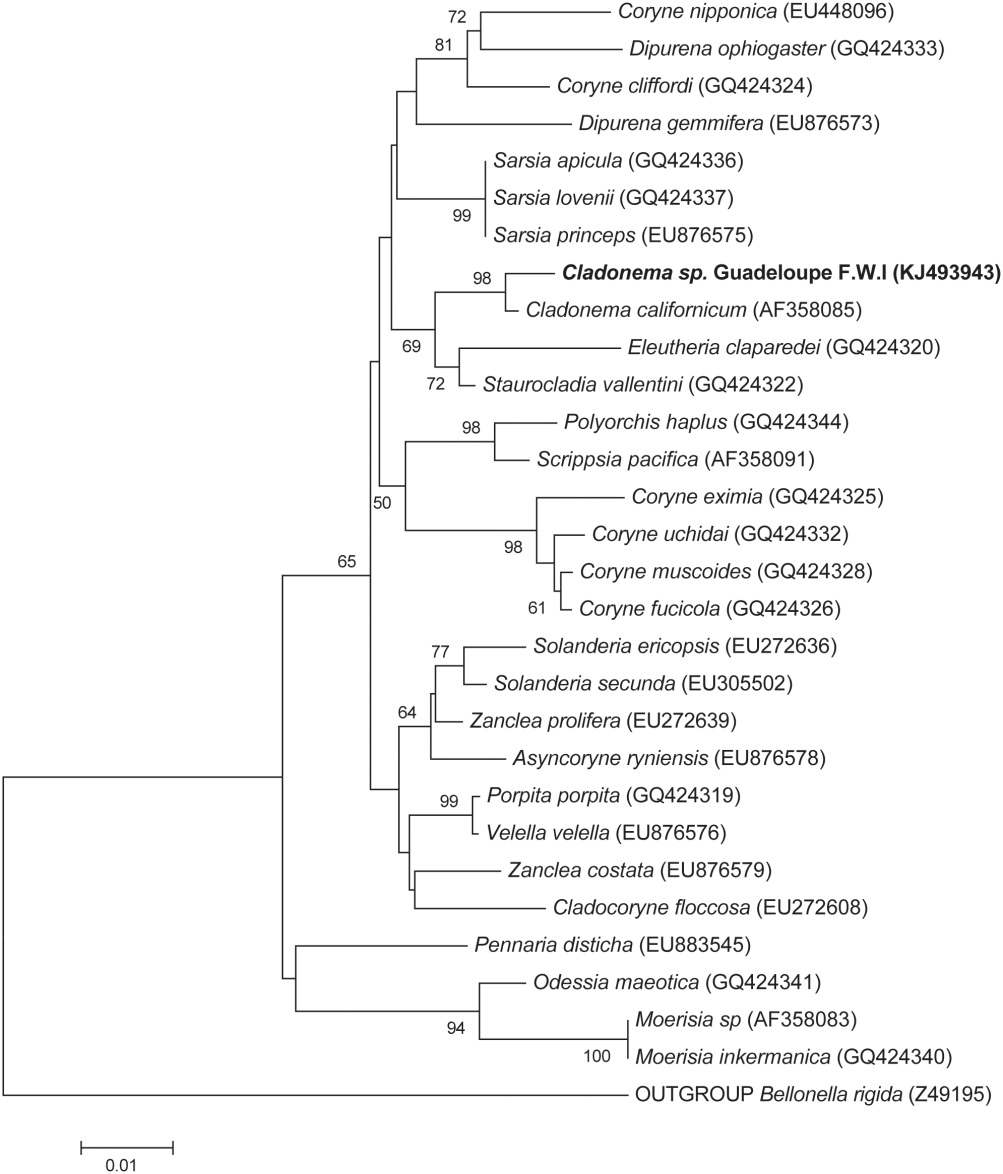
Phylogenetic tree. Maximum Likelihood tree displaying the phylogenetic relationships between *Cladonema* sp. Guadeloupe F.W.I. (**in bold**) with other capitata hydrozoans based on the analysis of partial 18S rRNA gene sequences of 895 nucleotides. *Bellonella rigida* was used as the outgroup. Only bootstrap values of more than 60% are shown at each node. The scale bar corresponds to 0.01 changes per nucleotide.

The phylogenetic reconstruction based on partial sequences of the genes coding for 18S rRNA, placed this organism on a capitata group of hydrozoan class (phylum Cnidaria). Maximum Likelihood (ML) tree revealed that this organism formed a distinct clade with the closest relative *Cladonema californicum* (Phylum: Cnidaria; class: Hydrozoa, order: Anthoathecata, suborder: Capitata, Family Cladonematidae, Genus: *Cladonema* sp.) (Fig 3) [7]. The sister group was supported by robust branch of the phylogenetic tree (98% bootstrap support from 1000 replicates). The sequences of the genes coding for 18S rRNA of these two *Cladonema* spp. differed by four bases and shared 99% pairwise identity suggesting that they could be the same species. However, the partial sequences obtained, and the lack of other sequences of the genes encoding 18S rRNA from *Cladonema* sp., did not allow us to confirm the relationship between these two hydrozoans. Further molecular investigations involving additional marker genes (i.e. COI, 28S rDNA and ITS) are needed in order to resolve in depth the phylogeny of these species.

#### Polyp *Cladonema* sp. Ectosymbionts

**Fig 4:**
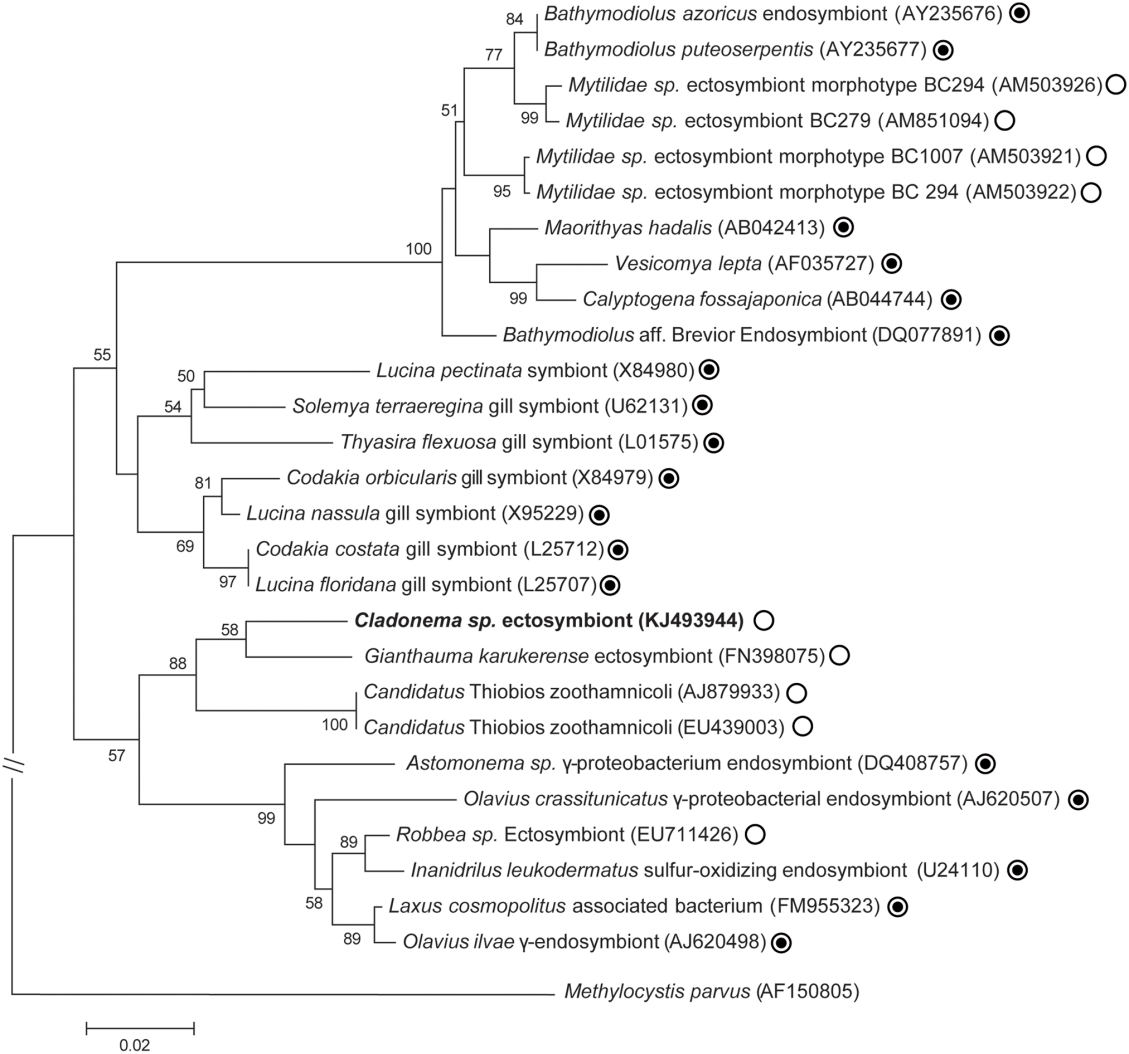
Phylogenetic tree. Maximum Likelihood tree displaying the phylogenetic relationships between the *Cladonema* sp. ectosymbiont (**in bold**) with other endo and ectosymbionts sulphur-oxidizing bacteria based on the analysis of 16S rRNA gene sequences of 926 nucleotides. *Methylocystis parvus* was used as the outgroup. Only bootstrap values of more than 50% are shown at each node. The white circles with or without black dot indicate whether symbionts are intra- or extracellular respectively. The scale bar corresponds to 0.02 changes per nucleotide.

A phylogenetic tree based on partial sequences coding for 16S rRNA genes, revealed that these bacteria belong to gamma-proteobacteria (Fig 4). Maximum likelihood (ML) tree showed that they were in a monophyletic group formed by other ectosymbiotic thioautotrophic bacteria retrieved from sulfide-rich environment. However, the percentage pairwise identity between *Cladonema* ectosymbiont and its closest relatives bacterial ectosymbiont species colonizing the giant archaea *Giganthauma karukerense* [61] is 95.3%, shows that it could be two different bacterial species. On the other hand, due to the very low individuals available for this study, it was unfortunately not possible to preserve polyps in PFA for *Fluorescent In Situ Hybridization* experiment with a specific probe designed from this sequence in order to check that such sequence corresponded to the ectosymbionts. However, the 16S rRNA gene sequence obtained allow to design two specific primers in order to amplify more specifically the ectosymbiont rRNA gene sequences from other polyps or from medusa.

#### Identification of *Cladonema* sp. medusa

**Fig 5:**
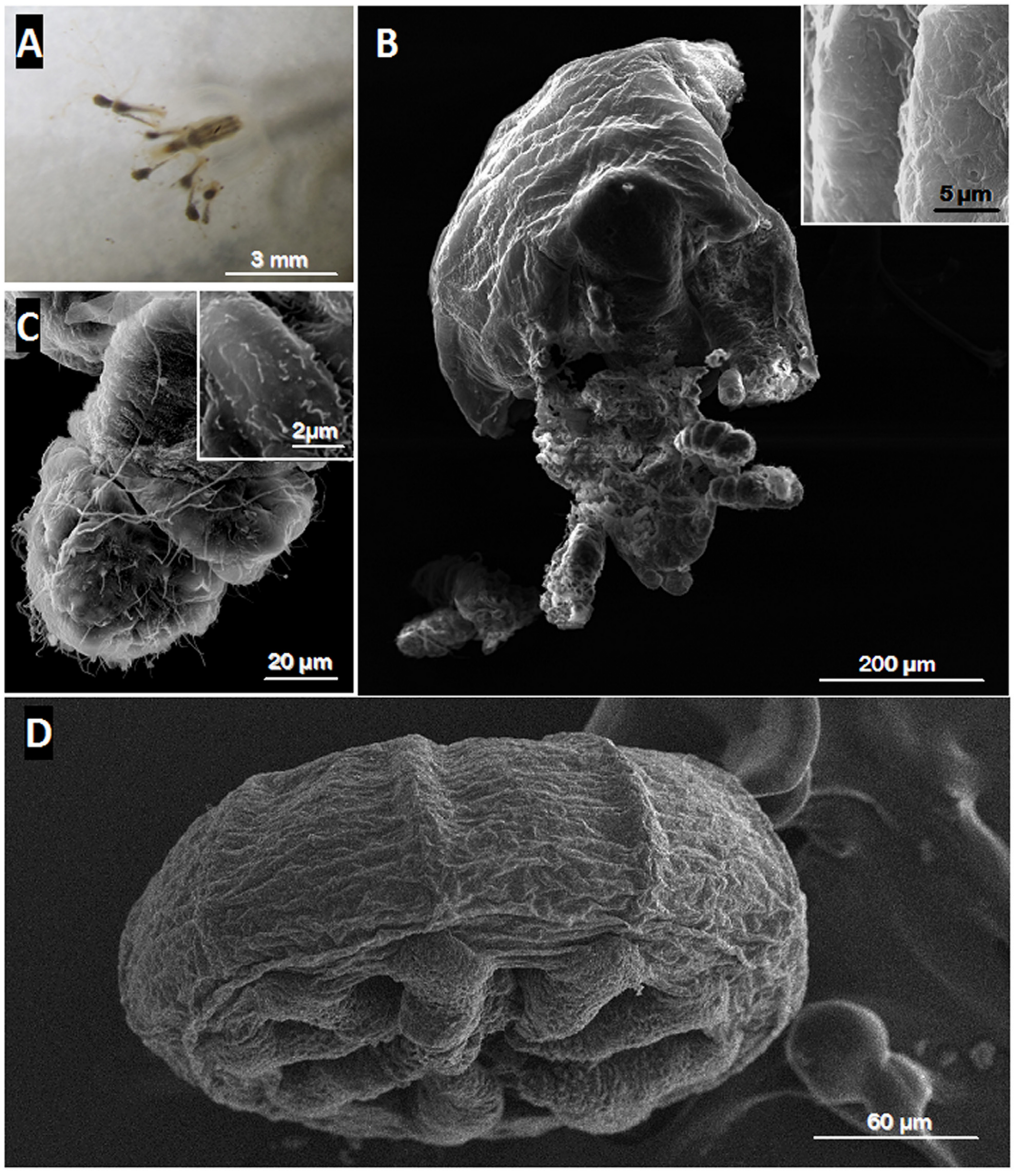
Ultrastructural analysis of a Medusa. Light micrograph of the medusoid stage of *Cladonema* sp. (A) shows that the tentacles extended around the bell while SEM image (D) shows that the tentacles were retracted due to the chemical fixation artefact. No bacterium was observed on the bell surface of the medusa (B, D) or on its tentacles (C-D). The two insets clearly confirmed the absence of ectosymbiotic bacteria on the bell surface (B) and on the tentacles (C) of the medusa.

Single medusa collected from the sea grass beds was small (around 3 mm length) and moving by pulsating the bell (Fig 5A). The sequence of partial 18S rRNA gene from medusa, showed 100% identity with the partial 18S rRNA gene sequence of the current polyps suggesting that the medusa and the polyps were the same animals but at two different stages. Conversely to the polyp stage, no bacteria were detected on the surface of the medusa either on the bell or on the tentacles (Fig 5B–5D). The surface of the tentacle contained few short cilia (Fig 5C) which are not to be confused with bacterial symbionts (Fig 1E was taken at the same magnification as Fig 5C). Thus, no bacterial ectosymbiont was observed on the medusa. Furthermore, the sulphur-oxidizing bacterial symbiont 16S rRNA gene could not be amplified by PCR from the medusa DNA using the specific primer set designed in this study (S1 Fig). Only a non-specific band (above the expected size) was obtained from the medusa DNA template confirming that no ectosymbiont was present in/on *Cladonema* sp. medusa. This PCR reaction result was consistent with the analysis of the medusa ultrastructure described above. Consequently, both results appear to indicate that the *Cladonema* sp. medusa was free of sulphur-oxidizing bacterial ectosymbionts.

## Discussion

Here we report on a thiotrophic symbiosis occurring between a cnidarian and sulphur-oxidizing bacteria in the sulfidic mangrove of Guadeloupe. Primary inspections, through closer morphological and ultrastructural examinations, unveiled the presence of prokaryote populations bearing elemental sulphur granules, thriving on the body surface of the metazoan species. Phylogenetic assessments were undertaken in order to identify the nature of the invertebrate and microorganisms involved in thiotrophic symbiosis. The results showed, with strong support, that this animal was closely related to the hydrozoan *Cladonema californicum* belonging to the class Hydrozoa and to the genus *Cladonema*, hence this metazoan species is named *Cladonema* sp. Phylogenetic analysis of the symbiotic bacteria attached to the cnidarian, assigned them within a well-supported clade formed by the sulphur-oxidizing epibionts, *Candidatus* Giganthauma karukerense [61] and *Candidatus* Thiobios zoothamnicoli [62] bacteria. Therefore, the *Cladonema* sp. bacterial ectosymbionts were sulphur-oxidizers, confirming previous observations made by ultrastructural analysis. It should be stressed that both ectosymbiotic bacteria mentioned above [61, 62], similar to the *Cladonema* sp. bacterial symbionts, were also found in the sulfidic mangrove of Guadeloupe. Taken together, the results from an attentive examination of this metazoan specimen, brought to light a novel kind of invertebrate-bacterial symbiosis, occurring between the hydrozoan *Cladonema* sp. belonging to the phylum Cnidaria and thioautotrophic bacteria.

### Why is this unique?

In the literature, articles describe cnidarians mostly as marine invertebrates harbouring photoautotrophic endosymbionts [2, 16–30]. Those that do mention the occurrence of thiobacteria in association with some members of the phylum Cnidaria, do not demonstrate the existence of mutualistic relationship [23, 29, 63–70]. More importantly, we have never come across any description of sulphur-oxidizers in ectosymbiosis with a cnidarian, after reviewing published literature on this subject.

### Polyps and medusa are two stages of the same animal

We could not attest via phylogenetic assessment that the polyp specimen was from the same species as *C*. *californicum*. However information collected on the latter has enable us to better define the metazoan *Cladonema* sp. Indeed, *C*. *californicum* has been reported to exhibit two adult morphologies, the polyp and semi benthic medusa during its life cycle [7, 71–74]. Moreover, Rees [75] studied *C*. *californicum* in greater details and represented it on a sketch with four capitate tentacles around the oral end of hydranths and four to five filiform tentacles located at the mature polyp base. Importantly, the author also indicated, the occurrence of nascent medusa through budding at the mid-region of hydroids, between the two rings of tentacles, a tissue mass area so-called entocodon. Surprisingly, similar features were observed on the SEM microphotographs taken from the polyps collected in sulphidic shallow-water environments of marine mangrove. Particularly, one could picture on these micrographs, entocodons budding laterally from the hydranth, a region between the two crowns of tentacles [9]. From this observation, we reasoned that *Cladonema* sp. collected, could equally exhibit two adult morphologies in its life cycle, namely medusae and polyps. Consequently, to find out whether this mangrove cnidarian produced the medusa adult form, we inspected areas close to the location where *Cladonema* sp. polyps were harvested. The analysis of the partial 18S rRNA gene sequences extracted from medusae, showed 100% identity with the polyp sequence, confirming our hypothesis. Hence, the polyp and the medusa were two distinct stages of the same animal, *Cladonema* sp., the latter cycling between these two adult forms.

### Distinct symbiotic status between medusae and polyps

The examinations of the polyp form of *Cladonema* sp. showed the occurrence of a symbiosis with sulphur-oxidizing bacteria. To determine the symbiotic status of the medusa, we looked through ultrastructural observations and via 16S rRNA gene sequence analysis of the *Cladonema* sp. symbiont from DNA extracted from the medusa sample. Both analyses were negative; no sulphur-oxidizing bacteria were present on the body of the medusa. Consistent with this observation, partial 16S rRNA *Cladonema* sp. symbiont gene sequences from the mixture of medusa, also failed to amplify. Interestingly, only the polyp stage of *Cladonema* sp. harboured the thioautotrophic ectosymbionts.

**Fig 6:**
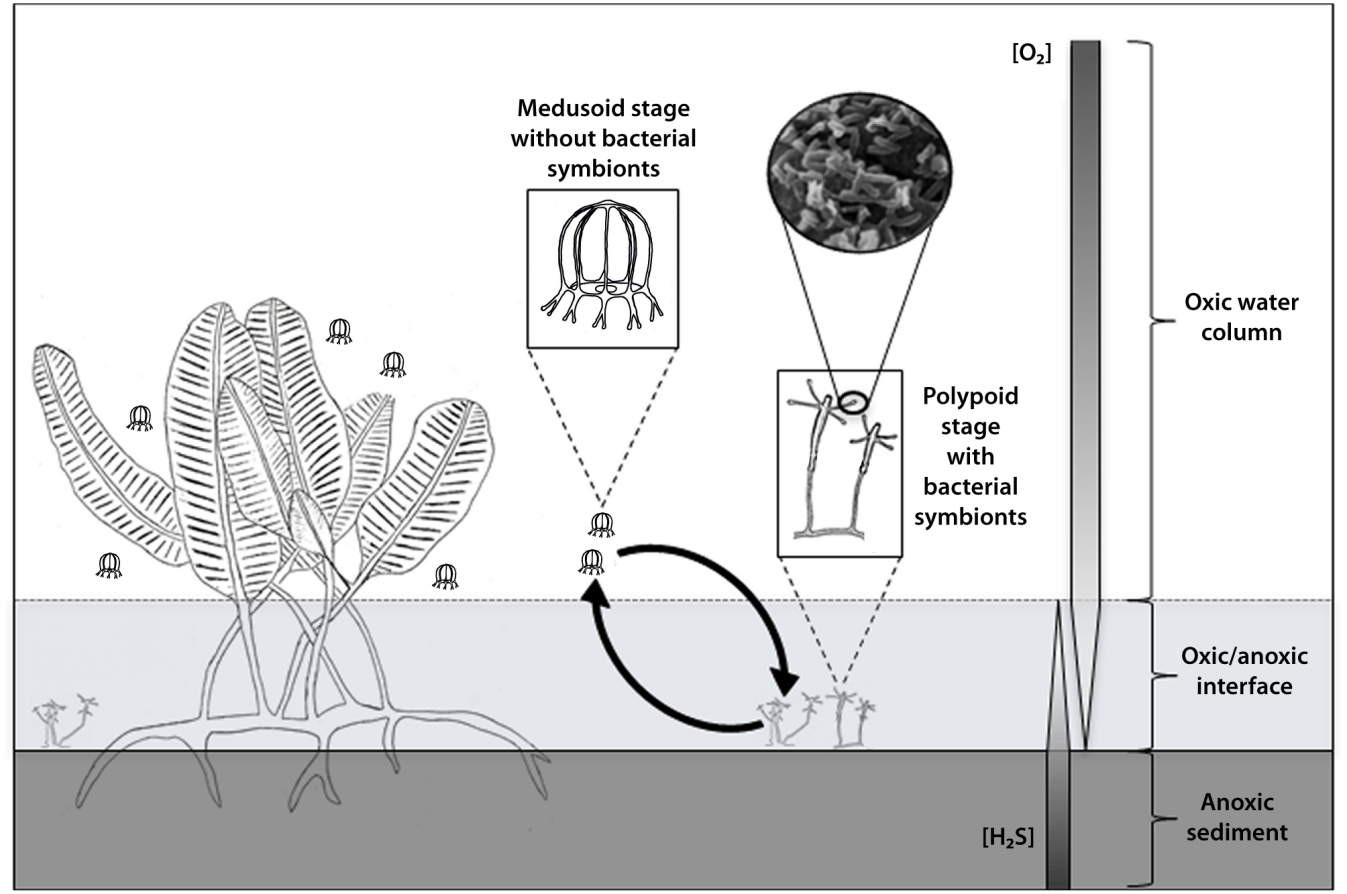
Symbiotic status model in *Cladonema* sp. The diagram depicts a model to explain the distinctive symbiotic status existing between polyps and medusae of *Cladonema* sp. which seems to rely on an inverse gradient of sulphur and oxygen in the environment. The drawing shows that only the polypoid stage of the cnidarian living in a sulphide rich environment (at the oxic/anoxic interface) bears symbiotic sulphur-oxidizing bacteria while the medusoid stage located in the water column depleted in sulphide, is free of such ectosymbiosis. Such medusa were usually observed closed to sea grasses of *Caulerpa taxifolia* within the mangrove lagoon.

To understand this behavioural dissimilarity between medusa and polyps in the face of the sulphur-oxidizing symbionts, we looked into the sulphur-oxidizer requirements. A fundamental requirement is the presence of an inverse gradient of hydrogen sulphide and oxygen, so-called chemocline [36]. More precisely, it is at this anoxic-oxic interface which is enriched in sulphides that thioautotrophic symbioses develop [32–34]. Sulfidic concentration in the mangrove swamp in which our samples were collected, has been measured in the past by Laurent et *al*. and Muller et *al*. [50, 61]. They showed that the sulphide concentration was lower in the mangrove water column with a concentration of 0.1 μM while in the mangrove sediment, it could reach up to 1mM, thus, validating the high content of sulphide in the mangrove sediment. When we analysed with caution the locations in which medusa and polyps were harvested, it was clear-cut that the sulfidic status of these two sites were divergent. The polyps were found tethered on mangle leaves lying on the mangrove sediment rich in sulphide. In stark contrast, the medusae were living in the sea grass bed of the mangrove water column depleted in sulphide. Thus, only the polypoid stage of the cnidarian, could harbour the thioautotrophic ectosymbionts as they live in a sulphidic rich environment. Fig 6 illustrates the distinctive symbiotic status existing between these two forms that clearly relies on an inverse gradient of sulphur and oxygen present in the mangrove ecosystem. It is possible that the immature medusae can actually shelter thioautotrophic ectosymbionts when still attached to the hydroid, but we did not observe such association on the surface of the polyp entocodon.

### Benefit of such symbiosis

Symbiosis when beneficial to both partners (mutualism) can provide them with new capabilities. In this respect, nourishment either through metabolic exchanges [2, 16–30] or digestion of symbiotic microorganisms by the host [76–78] has been proven to be an effective way to sustain organisms in environment deprived in nutrients. Yet, symbiosis also takes part in a range of processes very diverse such as defence against predator as reported in bioluminescent symbiosis in the squid *Euprymna scolopes* and *Vibrio fisheri* [79, 80] or animal ontogeny and reproduction, in biology, as reviewed by McFall-Ngai *et al*. [81].

In the case of sulphur-oxidizing bacterial symbiosis, in addition to nutritional exchanges occurring between these symbionts and marine invertebrates inhabiting sulfidic habitats, other important roles have been documented. One involves the putative detoxification of the host environment by these thiobacteria. Indeed, chemoautotrophic symbionts use reduced compounds (such as HS^-^ in the environment) to yield energy in order to supply organic carbon to their metazoan hosts [32, 34]. This process may contribute to reducing the amount of these toxic compounds in the vicinity of the host. Minimizing sulfide toxicity, has been proposed for some symbiotic sulfide-oxidizing microorganisms [82, 83] enabling the hosts to colonize ecological niches which are in fact toxic for most of the sessile eukaryotic organisms.

With regards to *Cladonema* sp., only its benthic form, the polyp living in rich sulfidic environment, harbours sulphur-oxidizing bacteria. These findings, in the light of what is known about sulphur-oxidizing symbioses [82–84], suggests that sulphur-oxidizing ectosymbionts could assist the host *Cladonema* sp. polyp in this extreme environment, in terms of both nutrition and detoxification of the milieu. For example, it is well-known that HS^-^ is an inhibitor of the respiratory chain, so it is plausible that the ectosymbionts might assist the polyp to reduce the sulphide toxic effect in its microenvironment. However, in *N*. *ictus* and *N*. *frasassianus* amphipods, sulfide-oxidizing thiothrix ectosymbionts do not play an active role in sulfide detoxification of the host environment [42]. Other possibilities that could explain such symbiosis, could relate to nutrition such as nutritional exchanges between these two partners, or simply grazing. Metabolic transfers were suggested between chemosynthetic epibionts and the shrimp *Rimicaris exoculata*, but the identity of such organic compounds transferred remains elusive [84]. More studies need to be carried out to determine the role that these sulphur-oxidizing ectosymbionts play in *Cladonema* sp. polyps such as amplification of functional genes involved in sulphur metabolism (as *aprA*, *sox*, etc.) or nanosims experiments using ^13^C labelled CO_2_ in order to clarify the carbon transfer to the host tissues via bacterial CO_2_ fixation. The main problem for such additional experiments will be to find more polyps as they are difficult to collect and consequently quite rare to investigate.

In summary, to the best of our knowledge, this is the first report to date on a thiotrophic symbiosis involving a cnidarian. This study is a jump-start to carry out more investigations in order to decipher the molecular pathways involved in the sulphide rich habitat adaptations in sulphur-oxidizing ectosymbioses.

## Supporting Information

S1 Fig PCR amplifications of *Cladonema* sp. polyp ectosymbiont. The PCR amplifications of *Cladonema* sp. polyp ectosymbiont were done using the specific primer set designed in this study. The gel shows specific DNA bands representing a 440bp region of sulphur-oxidizing symbiont 16S rRNA gene in lanes 5 and 8 accordingly to phylogenetic analysis. Lane 1: ladder, lane 2: negative control (H_2_O), lane 3: *Escherichia coli* DNA, lane 4: *Cyanobacterium* sp. DNA, lane 5: *Zoothamnium* sp. DNA, lane 6: *Zoothamnium* sp. DNA dilution 1/10, lane 7: *Cladonema* sp. medusa DNA, and lane 8: *Cladonema* sp. polyp DNA.
(TIF)
