# Supplementary figures and images for: First Description of Sulphur-Oxidizing Bacterial Symbiosis in a Cnidarian (Medusozoa) Living in Sulphidic Shallow-Water Environments
Source: PLoS One. 2015 May 26;10(5):e0127625. doi: 10.1371/journal.pone.0127625 (PMC4444309; doi:10.1371/journal.pone.0127625)

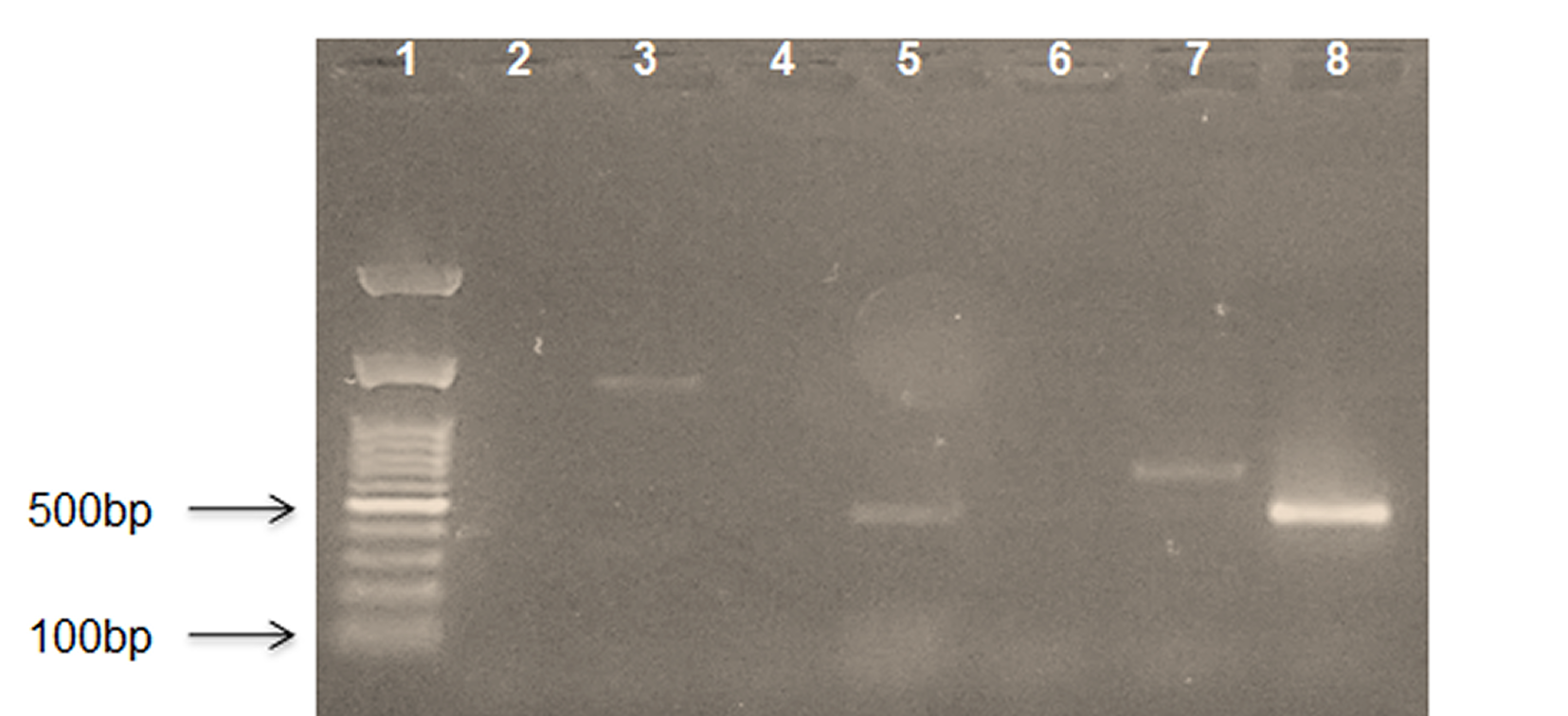

Supplement: S1 Fig — The PCR amplifications of Cladonema sp. polyp ectosymbiont were done using the specific primer set designed in this study. The gel shows specific DNA bands representing a 440bp region of sulphur-oxidizing symbiont 16S rRNA gene in lanes 5 and 8 accordingly to phylogenetic analysis. Lane 1: ladder, lane 2: negative control (H2O), lane 3: Escherichia coli DNA, lane 4: Cyanobacterium sp. DNA, lane 5: Zoothamnium sp. DNA, lane 6: Zoothamnium sp. DNA dilution 1/10, lane 7: Cladonema sp. medusa DNA, and lane 8: Cladonema sp. polyp DNA. (TIF) [file pone.0127625.s001.tif]
